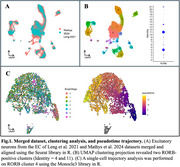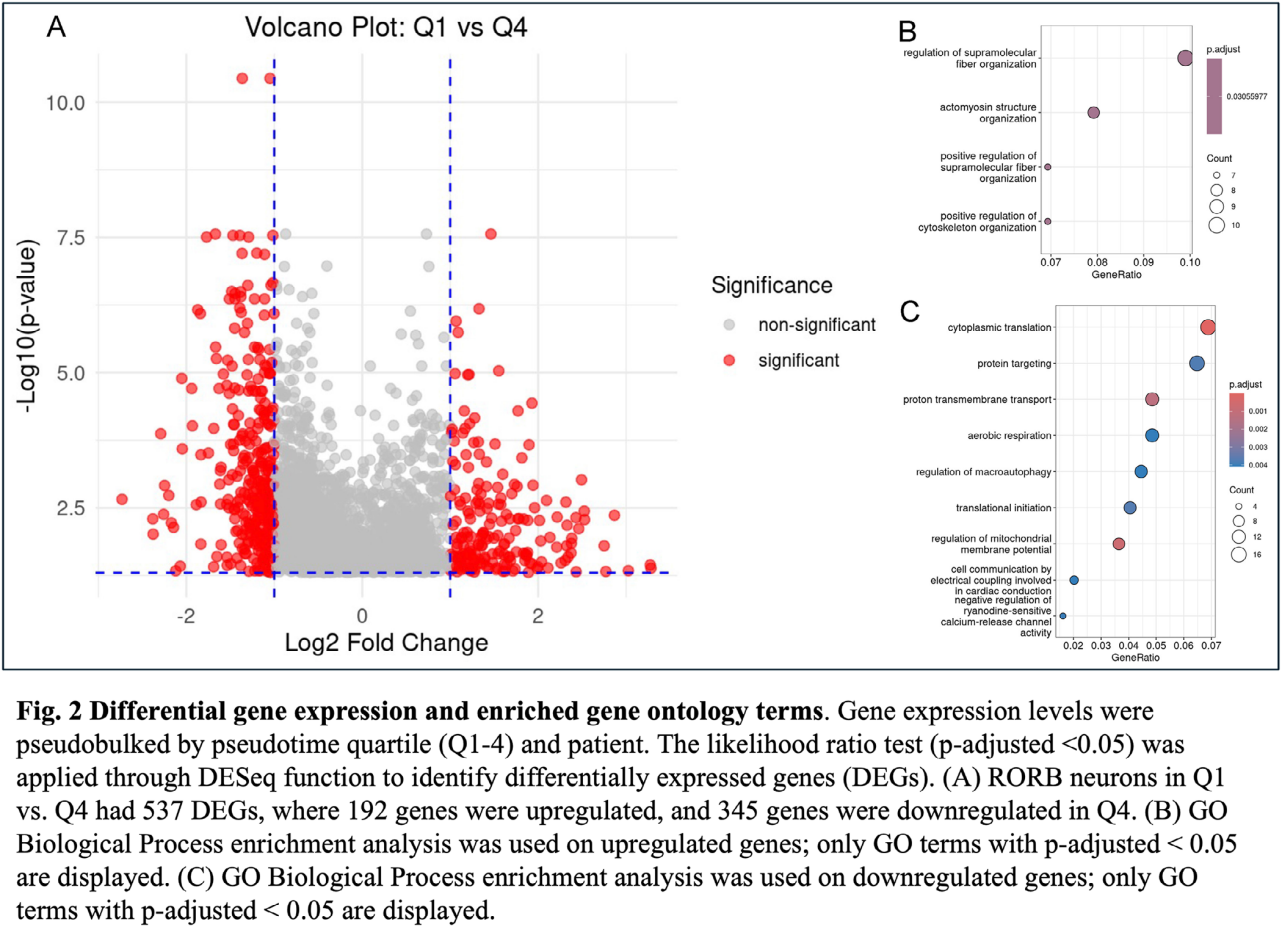# Exploring molecular signatures of vulnerable vs resilient RORB excitatory neurons of the entorhinal cortex in Alzheimer's disease

**DOI:** 10.1002/alz70855_107472

**Published:** 2025-12-25

**Authors:** Alexander V. Soloviev, Felipe Luiz Pereira, Renata Elaine Paraizo Leite, Claudia Kimie Suemoto, Kun Leng, Martin Kampmann, Lea T. Grinberg

**Affiliations:** ^1^ Memory and Aging Center, UCSF Weill Institute for Neurosciences, University of California, San Francisco, San Francisco, CA, USA; ^2^ Department of Pathology, University of São Paulo Medical School, São Paulo, São Paulo, Brazil; ^3^ Physiopathology in Aging Laboratory (LIM‐22), Department of Internal Medicine, University of São Paulo Medical School, São Paulo, São Paulo, Brazil; ^4^ Division of Geriatrics, Department of Internal Medicine, University of São Paulo Medical School, São Paulo, São Paulo, Brazil; ^5^ Medical Scientist Training Program, University of California San Francisco, San Francisco, CA, USA; ^6^ Department of Biochemistry and Biophysics, Weill Institute for Neurosciences, University of California San Francisco, San Francisco, CA, USA

## Abstract

**Background:**

Identifying the molecular mechanisms underlying selective neuronal vulnerability and resilience is crucial for developing effective treatments for Alzheimer's disease (AD). Our group has shown that RORB‐positive excitatory neurons are selectively vulnerable and preferentially accumulate *p*‐tau inclusions in the entorhinal cortex (EC) at early Braak stages. However, not all RORB‐positive neurons are vulnerable. By leveraging single‐nucleus RNA sequencing (snRNA‐seq) data, we aim to identify the molecular pathways that differentiate vulnerable and resilient RORB subtypes. We hypothesize that these biological differences may be key to understanding the mechanisms of selective neuronal vulnerability in AD.

**Method:**

We analyzed two snRNA‐seq datasets of isolated nuclei extracted from the EC of postmortem brain tissue from healthy controls and AD patients (Leng et al. 2021 [*n* = 10] and Mathys et al. 2024 [*n* = 48]). Focusing on RORB neurons, Monocle3 trajectory analysis mapped cell state progression across Braak stages. In neurons from Braaks 0‐2, we performed pseudobulk and DESeq2 analysis on the first (Q1) and fourth (Q4) quartiles of pseudotime to identify differentially expressed genes (DEGs). Q1 (pseudotime = 0‐5) represented resilient/healthy transcriptomes, while Q4 (pseudotime = 15‐20) represented vulnerable/stressed transcriptomes.

**Result:**

After merging datasets and subsetting excitatory neuronal nuclei (*n* = 53284), UMAP clustering identified two RORB‐positive populations in the EC (Figure 1A and B). Within a RORB population (cluster 4), the trajectory analysis revealed a progression of cell states represented by an increase in pseudotime (Figure 1C). Preliminary results comparing resilient and vulnerable RORB molecular signatures revealed 537 DEGs (Figure 2A). Vulnerable neurons significantly upregulated pathways related to supramolecular fiber organization and downregulated pathways related to protein translation, targeting, and quality control (Figure 2B and C).

**Conclusion:**

Our findings suggest that transcriptomic signatures of vulnerable and resilient RORB excitatory neurons in the EC, in early Braak stages, may reveal key pathways leading to tau accumulation and neuronal loss. Notably, vulnerable RORB neurons upregulated cytoskeletal organization pathways and downregulated proteostasis pathways. Ongoing analyses on this RORB subpopulation and trajectory analyses on additional excitatory neuronal subpopulations in the EC will refine our understanding of the pathways that potentially lead to selective vulnerability in AD.